# Ginsenoside Rg3 Attenuates Angiotensin II-Mediated Renal Injury in Rats and Mice by Upregulating Angiotensin-Converting Enzyme 2 in the Renal Tissue

**DOI:** 10.1155/2019/6741057

**Published:** 2019-11-29

**Authors:** Hui Liu, Yichuan Jiang, Min Li, Xiaofeng Yu, Dayun Sui, Li Fu

**Affiliations:** ^1^Department of Pharmacology, School of Pharmaceutical Sciences, Jilin University, Changchun 130021, China; ^2^Jilin Yatai Pharmaceutical Co., Ltd., Changchun 130033, China; ^3^Institute of Dalian Fusheng Natural Medicine, Dalian 116600, China

## Abstract

Angiotensin II- (Ang II-) mediated renal injury represents a major pathogenetic mechanism in most chronic kidney diseases. Our previous research demonstrated that ginsenoside Rg3 (Rg3) attenuates Ang II elevation in the myocardium in spontaneously hypertensive rats (SHR). It is possible that Rg3 has similar effects in the renal tissue. In this research, we first demonstrated that Rg3 could attenuate Ang II increase in the kidney of SHR and reduce hypertensive nephropathy progression. Then, we found that Rg3 attenuated Ang II increase by upregulating angiotensin-converting enzyme 2 (ACE2) in the renal tissue. We confirmed this finding in an exogenous Ang II-infused mice model of renal injury, and two models showed consistent results. In conclusion, Rg3 attenuates Ang II-mediated renal injury in rats and mice by upregulating ACE2 in the renal tissue. This research is the first to demonstrate that Rg3 increases tissue ACE2 levels *in vivo*.

## 1. Introduction

Ginsenoside Rg3 (Rg3), an uncommon ginsenoside transformed from other ginsenosides during heating [1], shows strong antitumor activity [2–6]. In the industrialized production, chemical [1] or biological [7, 8] techniques are employed for Rg3 synthesis. Shenyi Capsule, whose principal component is Rg3, constitutes a common antitumor drug in China, produced by our company (Jilin Yatai Pharmaceutical Co., Ltd.).

Besides its antitumor activity, Rg3 has also been reported to have cardiovascular protective effects through multiple mechanisms [6, 9, 10], including antiinflammation, antioxidative stress, and antifibrosis. Our study indicated that Rg3 exerts cardioprotective effects not depending on blood pressure reduction in spontaneously hypertensive rats (SHR), possibly in association with attenuated angiotensin II (Ang II) increase in the myocardium [10]. It is also known that Ang II increase mediates inflammation, oxidative stress, and fibrosis in the myocardium [11], which could be attenuated by Rg3.

Indeed, elevated Ang II induces not only cardiovascular injury but also kidney damage in SHR [12]. Hypertensive nephropathy represents a common complication of hypertension, and one of its major mechanisms is Ang II-associated inflammation, oxidative stress, and fibrosis in the renal tissue [13–15]. As the mechanisms of cardiovascular and renal injuries in SHR have common features, it is interesting to assess whether Rg3 has renal protective effects in SHR. Furthermore, if Rg3 has such effects in SHR and is associated with the attenuation of Ang II increase, an exogenous Ang II infusion mouse model of renal injury [15] could be used to further confirm these results.

## 2. Materials and Methods

### 2.1. Chemicals and Reagents

Rg3 (95% purity) provided by Jilin Yatai Pharmaceutical (China) was dissolved in 0.5% sodium carboxymethyl cellulose solution (0.5% CMC-Na) for use. Ang II (97% purity) was purchased from Sigma-Aldrich (USA) and dissolved in physiological saline. The remaining chemicals were of analytical grade.

### 2.2. Animals and Treatments

A total of 16 Wistar-Kyoto rats (WKY) and 16 SHR (male, 16∼17 weeks old) (Beijing Vital River Laboratory Animal Technology, China) were housed with rodent chow and water *ad libitum*. Experiments involving animals followed the Guide for the Care and Use of Laboratory Animals of Jilin University, with approval from the institutional Ethics Committee.

The animals were grouped into four: *WKY* group (8 WKY, orally administered 0.5% CMC-Na); *SHR* group (8 SHR, orally administered 0.5% CMC-Na); *WKY* + *Rg3* group (8 WKY, orally administered 20 mg·kg^−1^·d^−1^ Rg3); *SHR* + *Rg3* group (8 SHR, orally administered 20 mg·kg^−1^·d^−1^ Rg3).

Rg3 or placebo administration was carried out once daily for 42 days. This was followed by animal sacrifice and blood and renal tissue sample collection. The renal tissue specimens underwent fixation with 4% formalin (histopathology) or were snap-frozen with liquid nitrogen and kept at −80°C (reverse transcription quantitative real-time polymerase chain reaction (RT-qPCR) and enzyme-linked immunosorbent assay (ELISA)).

A total of 24 C57BL/6 mice (male, 10 weeks old) (Beijing Vital River Laboratory Animal Technology) were maintained with rodent chow and water at will. Experiments involving animals followed the Guide for the Care and Use of Laboratory Animals of Jilin University, with approval from the institutional Ethics Committee.

Subcutaneous implantation of a 1002 osmotic minipump (Alza, USA) was performed at the dorsum of the neck for Ang II (1.5 mg·kg^−1^·d^−1^) or normal saline infusion [15]. The animals were assigned to four groups: *Saline* group (4 mice, infused with normal saline and orally administered 0.5% CMC-Na); *Ang II* group (4 mice, infused with Ang II and orally administered 0.5% CMC-Na); *Saline* + *Rg3* group (4 mice, infused with normal saline and orally administered 20 mg·kg^−1^·d^−1^ Rg3); *Ang II* + *Rg3* group (4 mice, infused with Ang II and orally administered 20 mg·kg^−1^·d^−1^ Rg3).

Rg3 or placebo administration was performed daily for 14 days. This was followed by animal sacrifice and blood and renal tissue sample collection. The renal tissue specimens underwent fixation with 4% formalin (histopathology) or were snap-frozen with liquid nitrogen and kept at −80°C (RT-qPCR and ELISA).

### 2.3. Blood Pressure Assessment

Systolic (SBP) and diastolic (DBP) blood pressure measurements in rats and mice were performed by the tail-cuff technique using a small animal sphygmomanometer (BP-2010A; Softron Biotechnology, China) [16] on the initial and final days of treatment (6 and 2 weeks in rats and mice, respectively).

### 2.4. Serum Creatinine and Blood Urea Nitrogen (BUN) Level Assessment

Blood specimens were submitted to centrifugation (1500 g, 4°C for 15 min), and the resulting serum was kept at −80°C for biochemical assays. Creatinine assay and BUN assay kits were purchased form Nanjing Jiancheng Bioengineering Institute (China), and creatinine and BUN levels were assayed in accordance with the manufacturer's protocols.

### 2.5. Histopathological Assessment

Renal tissue specimens underwent fixation with 4% formalin, paraffin embedding, sectioning at 4 *μ*m, and staining with hematoxylin and eosin (H&E) and Masson trichrome stain, respectively. Then, a Nikon E100 light microscope (Nikon, Japan) was employed for analysis.

### 2.6. Immunohistochemistry (IHC)

Antiangiotensin-converting enzyme (ACE) and antiangiotensin-converting enzyme 2 (ACE2) primary antibodies were produced by Bioss Antibodies (China). Peroxidase-linked goat anti-rabbit secondary antibodies and the DAB and two-step rabbit IHC kits were provided by ZSGB-BIO (China). IHC was carried out as proposed by ZSGB-BIO. Photomicrographs were acquired and assessed by Image Pro Plus 6.0 (Media Cybernetics, USA).

### 2.7. Ang II and Angiotensin 1-7 (Ang 1-7) Level Assessment in the Renal Tissue

The supernatants of renal specimens were prepared as follows. A total of 100 mg of the renal tissue was homogenized in 900 *μ*L of ice-cold normal saline and submitted to centrifugation (1000 g, 4°C for 15 min). The resulting supernatants were kept at −80°C until analysis with Ang II and Ang 1-7 ELISA kits (Cusabio Biotech, China), respectively, in accordance with the manufacturer's protocols.

### 2.8. RNA Purification and RT-qPCR

Total RNA isolation was carried out with TRIzol reagent (Thermo Fisher Scientific, USA) as directed by the manufacturer. Reverse transcription and qPCR were carried out with TransScript Green Two-Step qRT-PCR SuperMix (TransGen Biotech, China) on a Stratagene Mx3000P (Agilent Technologies, USA) at 94°C (5 sec), 60°C (15 sec), and 72°C (10 sec) for 40 cycles. The 2^−ΔΔCt^ method [17] was employed for analysis, with GAPDH used for normalization. Primers are listed in Table 1.

### 2.9. Statistical Analysis

SPSS 16.0 (SPSS, USA) was employed for all statistical analyses. Data are mean ± standard deviation (SD). One-way analysis of variance (ANOVA) with Tukey's post hoc test was employed for group comparisons, with *P* < 0.05 indicating statistical significance.

## 3. Results

### 3.1. Rg3 Attenuates Early Nephropathy in SHR

**Figure 1:**
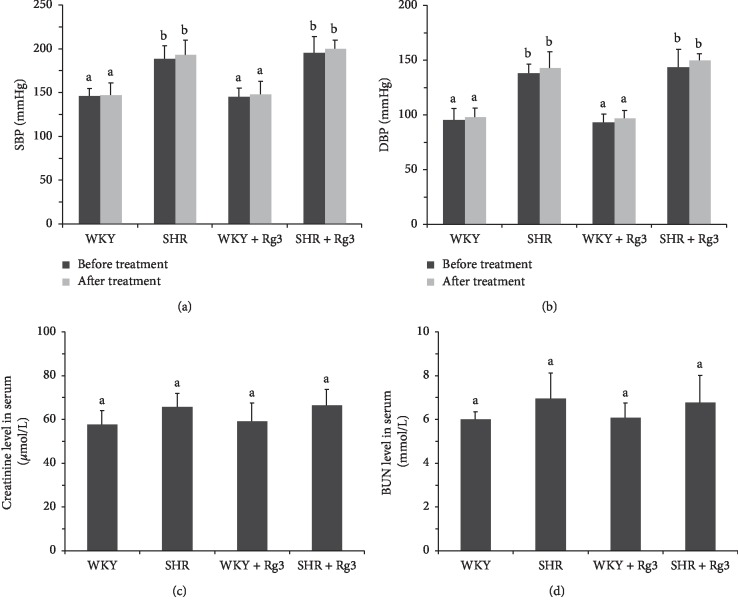
Blood pressure and serum markers of renal function in rats. SBP (a) and DBP (b) of rats prior to and following 6-week treatment; creatinine (c) and BUN (d) levels in serum in rats. Data are presented as the mean ± standard deviation, *n* = 8. The same superscript letters indicate no significant difference between groups (*P* > 0.05); significant difference existed between groups that do not have the same superscript letter (*P* < 0.05).

As we mentioned in a previous report [10], Rg3 had no significant effect on blood pressure. As shown in Figures 1(a) and 1(b), SBP and DBP in the two groups of SHR were markedly elevated in comparison with those of the two groups of WKY before treatment. Similar findings were obtained after the 6-week treatment. In addition, blood pressure in neither WKY nor SHR was changed by Rg3 treatment.

**Figure 2:**
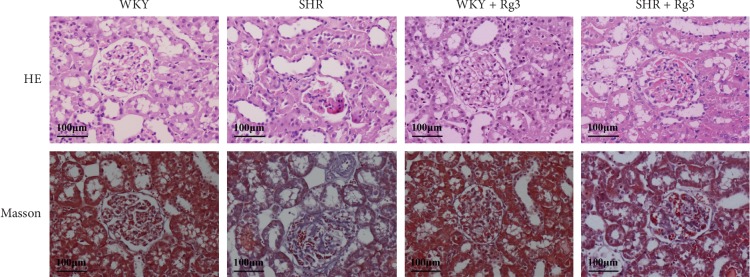
Representative HE and Masson staining histology photomicrographs of the renal tissue in rats.

Similar to the cardioprotective effects of Rg3 in SHR, Rg3 exerted renal protective effects independent of blood pressure reduction. Kidney H&E- and Masson-stained sections in the *WKY* + *Rg3* group showed the same features found in the *WKY* group. Renal sections of SHR showed congestion of glomerular capillaries and tubulointerstitial fibrosis based on Masson staining; Rg3 could attenuate these changes in glomerular capillaries and tubules (Figure 2).

The histopathological changes in kidneys of SHR were at the early stage in this study. This finding was confirmed by serum creatinine and BUN levels (Figures 1(c) and 1(d)). Creatinine and BUN levels in the two groups of SHR were somewhat elevated in comparison with those of the two groups of WKY, but the differences were not significant. The *SHR* and *SHR* + *Rg3* groups also showed comparable values.

These results indicated that hypertension-associated nephropathy in SHR was at the compensation stage. Rg3 could attenuate early nephropathy in SHR and might be able to prevent it from progressing into the decompensation stage.

### 3.2. Rg3 Reduces Ang II Levels in the Renal Tissue in SHR

There are two main angiotensin-converting enzymes which regulate the levels of Ang II *in vivo*, including ACE and ACE2 [18, 19]. ACE transforms angiotensin I into Ang II, and ACE2 transforms Ang II into Ang 1-7. So upregulation of ACE induces Ang II increase, while ACE2 upregulation attenuates Ang II increase.

**Figure 3:**
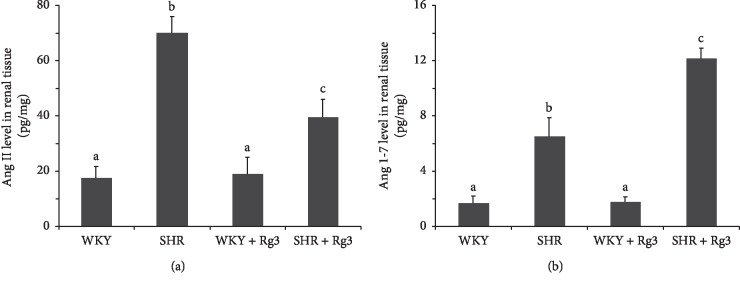
Angiotensin levels in the renal tissue in rats. (a) Ang II levels in renal tissue; (b) Ang 1-7 levels in renal tissue. Data are presented as the mean ± standard deviation, *n* = 8. The same superscript letters indicate no significant difference between groups (*P* > 0.05); significant difference existed between groups that do not have the same superscript letter (*P* < 0.05).

According to ELISA data (Figure 3), Ang II levels in the renal tissue of the SHR group were significantly higher than those of *WKY* and *WKY* + *Rg3* groups. Treatment with Rg3 significantly reduced Ang II levels in the renal tissue in SHR, while showing limited effects in the WKY group. Ang 1-7 amounts in the *SHR* group were significantly higher than those of the *WKY* and *WKY* + *Rg3* groups, with the *SHR* + *Rg3* showing even significantly higher values compared with the *SHR* group.

**Figure 4:**
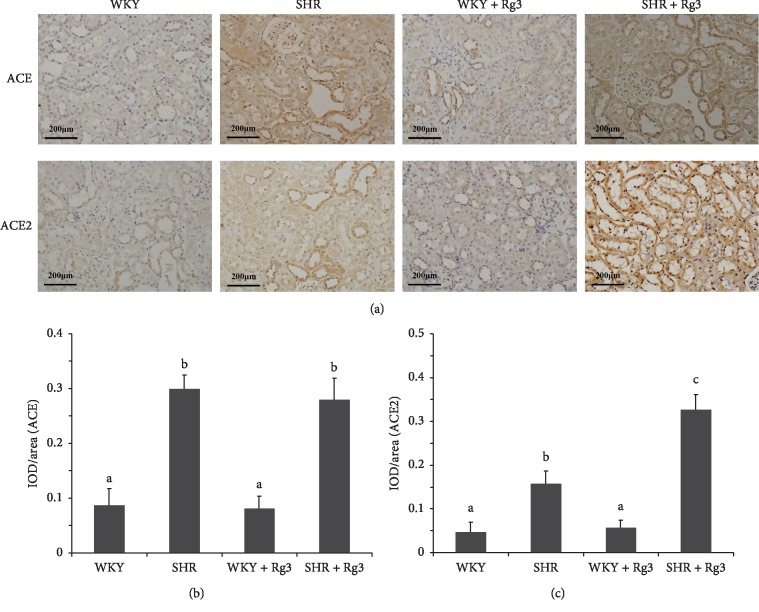
Levels of the angiotensin-converting enzymes in the renal tissue in rats. (a) Representative IHC staining photomicrographs of the renal tissue in rats. Quantitative results of IHC staining, which were presented as IOD/area and were proportional to the levels of ACE (b) and ACE2 (c). Data are presented as the mean ± standard deviation, *n* = 4. The same superscript letters indicate no significant difference between groups (*P* > 0.05); significant difference existed between groups that do not have the same superscript letter (*P* < 0.05).

IHC results (Figure 4) showed that ACE levels were markedly elevated in the two SHR groups compared with the two WKY groups. ACE2 amounts in the renal tissue were compensatorily upregulated in the *SHR* group, compared with the *WKY* and *WKY* + *Rg3* groups. Meanwhile, Rg3 treatment could further upregulate ACE2 in the renal tissue in SHR. This was the main mechanism that Rg3 treatment downregulated Ang II and upregulated Ang 1-7 in the kidneys of SHR. Meanwhile, Rg3 had no significant effect on ACE expression in either WKY or SHR.

### 3.3. Rg3 Attenuates Inflammation, Oxidative Stress, and Fibrosis in the Renal Tissue of SHR

It was reported that high Ang II levels in the renal tissue cause inflammation, oxidative stress, and fibrosis [15]. The mechanisms include inflammatory factor release, upregulated nicotinamide adenine dinucleotide phosphate (NADPH) oxidase, and activation of the TGF-*β*1/Smad signaling pathway. These changes also occurred in SHR.

**Figure 5:**
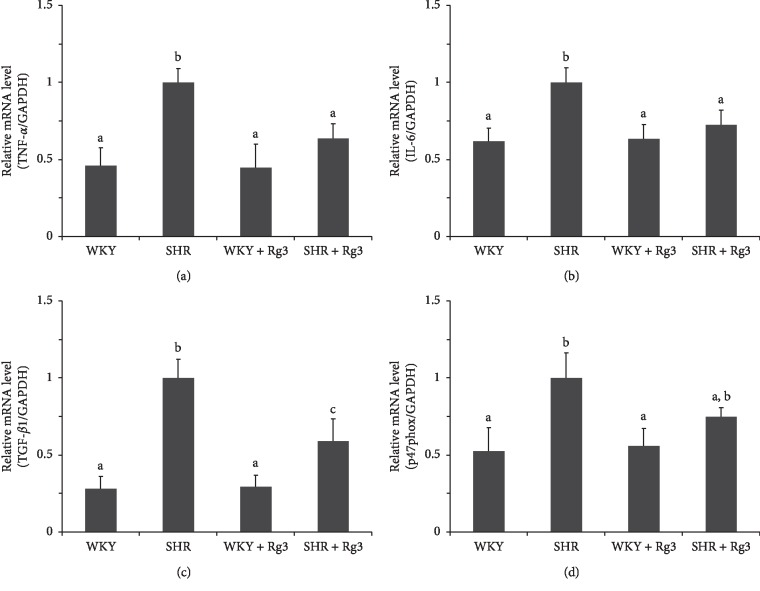
Levels of mRNA associated with inflammation, oxidative stress, and fibrosis in the renal tissue in rats. Relative mRNA levels of TNF-*α* (a), IL-6 (b), TGF-*β*1 (c), and p47phox (d). GAPDH was used as a housekeeping gene. Data are presented as the mean ± standard deviation, *n* = 4. The same superscript letters indicate no significant difference between groups (*P* > 0.05); significant difference existed between groups that do not have the same superscript letter (*P* < 0.05).

RT-qPCR data (Figure 5) demonstrated the relative mRNA amounts of TNF-*α*, IL-6, p47phox (NADPH oxidase subunits), and TGF-*β*1 in the renal tissue in the SHR group were remarkably elevated compared with those of the two WKY groups. The upregulation mediated by Ang II could be inhibited by Rg3 treatment, as the levels of these four mRNAs in the *SHR* + *Rg3* group were significantly lower than those of the *SHR* group, with no significant difference compared with the two WKY groups. These data indicated Rg3 treatment attenuated inflammation, oxidative stress, and fibrosis in the renal tissue in SHR.

### 3.4. Rg3 Attenuates Ang II-Mediated Kidney Injury in Mice

**Figure 6:**
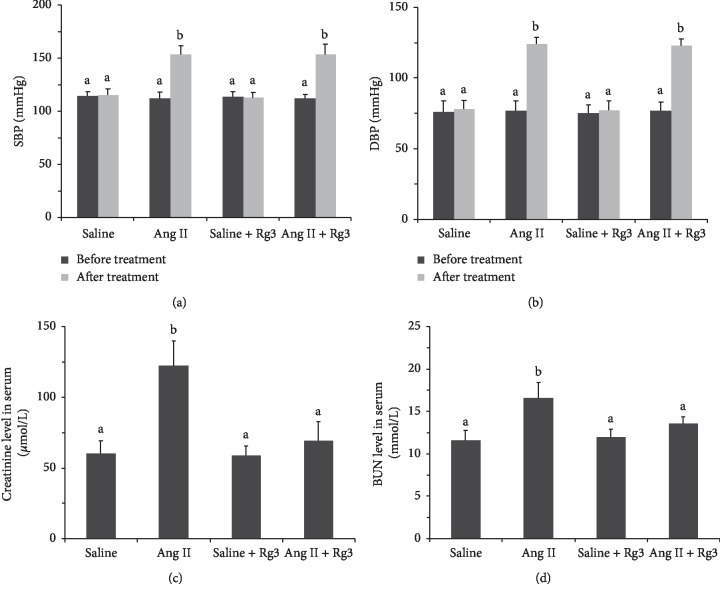
Blood pressure and serum markers of renal function in mice. SBP (a) and DBP (b) of mice prior to and following 2-week treatment; creatinine (c) and BUN (D) levels in serum in mice. Data are presented as the mean ± standard deviation, *n* = 4. The same superscript letters indicate no significant difference between groups (*P* > 0.05); significant difference existed between groups that do not have the same superscript letter (*P* < 0.05).

Ang II infusion by using the osmotic minipump (1.5 mg·kg^−1^·d^−1^) could raise blood pressure and mediate heart and kidney injury in C57BL/6 mice [11, 15]. In this research, SBP and DBP in the Ang II and Ang II + Rg3 groups were significantly higher than those of the other two groups after the 2-week Ang II infusion. With or without Ang II infusion, treatment with Rg3 did not change blood pressure in mice (Figures 6(a) and 6(b)).

**Figure 7:**
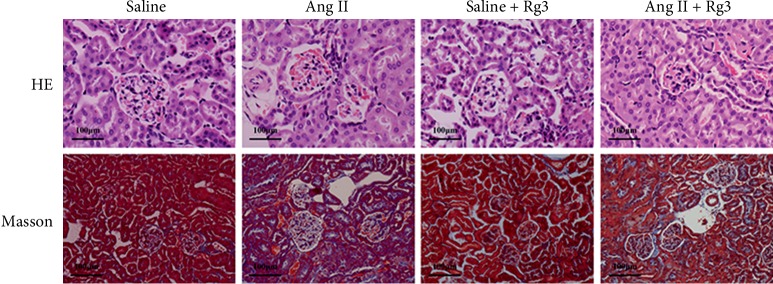
Representative HE and Masson staining histology photomicrographs of the renal tissue in mice.

According to H&E and Masson photomicrographs (Figure 7), histopathological changes in the kidneys of mice infused Ang II were more obvious compared with those of SHR; kidney sections showed glomerular changes, including hemorrhage, shrunken glomeruli, congestion of glomerular capillaries, and severe tubulointerstitial fibrosis in Masson staining. Rg3 could attenuate these changes in the kidney.

In accordance with histopathological data, creatinine and BUN levels in the *Ang II* group were markedly elevated compared with those of the other three groups, suggesting nephropathy in this group was decompensated. Creatinine and BUN levels in the other three groups had no significant differences, indicating Rg3 treatment improved renal function in mice infused Ang II, and prevented nephropathy progression from compensation to decompensation (Figures 6(c) and 6(d)).

### 3.5. Rg3 Reduces Ang II Levels in the Renal Tissue in Mice Infused Ang II

**Figure 8:**
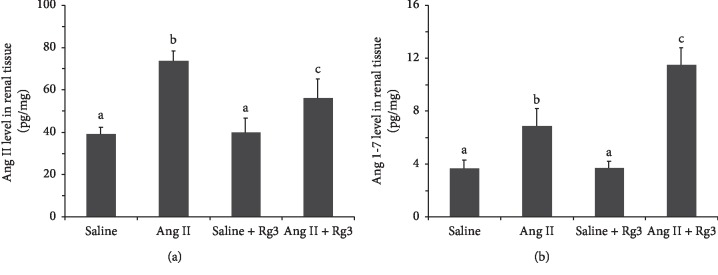
Angiotensins levels in the renal tissue in mice. Ang II (a) and Ang 1-7 (b) levels in the renal tissue. Data are presented as the mean ± standard deviation, *n* = 4. The same superscript letters indicate no significant difference between groups (*P* > 0.05); significant difference existed between groups that do not have the same superscript letter (*P* < 0.05).

According to ELISA data (Figure 8), Ang II infusion significantly raised Ang II levels in the renal tissue in mice, and Rg3 treatment could attenuate this increase significantly, although Ang II levels in the *Ang II* + *Rg3* group were still significantly higher than those of the two groups infused saline. Meanwhile, Ang 1-7 levels in the *Ang II* group were significantly higher than those of the *Saline* and *Saline* + *Rg3* groups; the *Ang II* + *Rg3* group showed a significant increase compared with the Ang *II* group.

**Figure 9:**
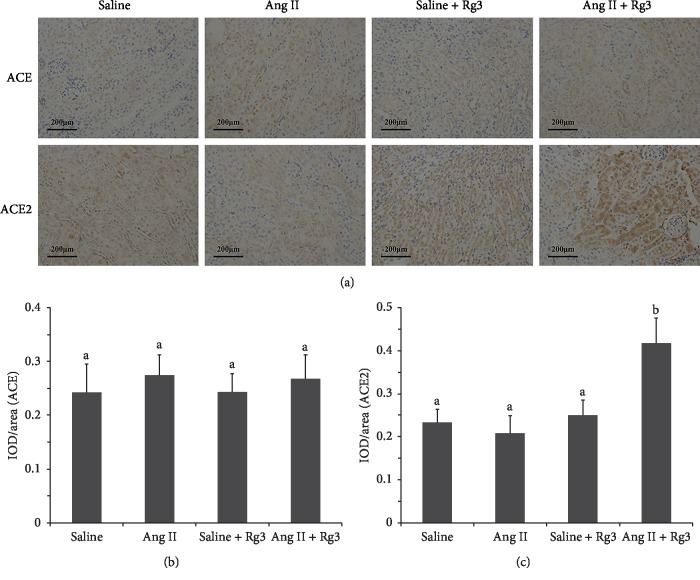
Levels of the angiotensin-converting enzymes in the renal tissue in mice. (a): Representative IHC staining photomicrographs of the renal tissue in mice. Quantitative results of IHC staining, which were presented as IOD/area and were proportional to the levels of ACE (b) and ACE2 (c). Data are presented as the mean ± standard deviation, *n* = 4. The same superscript letters indicate no significant difference between groups (*P* > 0.05); significant difference existed between groups that do not have a same superscript letter (*P* < 0.05).

IHC data (Figure 9) showed no marked differences in various group pairs in ACE expression. ACE2 expression levels in the *Ang II* + *Rg3* group were markedly elevated compared with those of the other three groups, which might be the main mechanism for degrading exogenous angiotensin.

### 3.6. Rg3 Attenuates Ang II Associated Inflammation, Oxidative Stress, and Fibrosis in Renal Tissue in Mice

**Figure 10:**
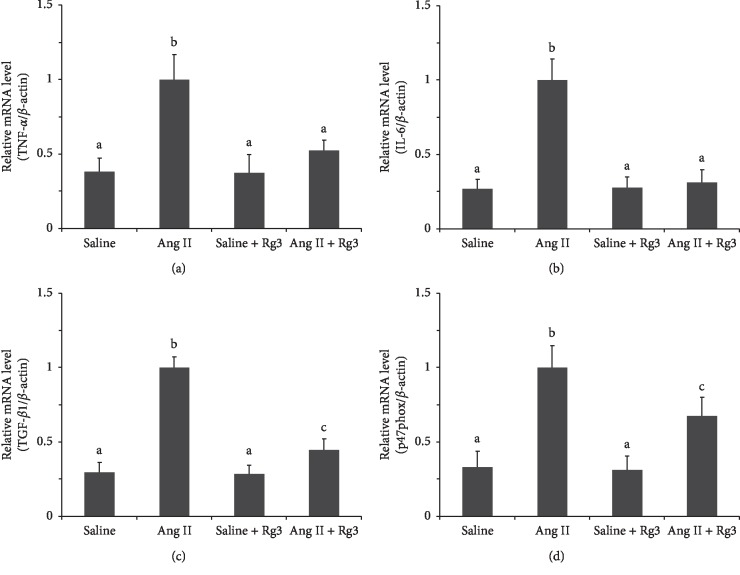
Levels of mRNA associated with inflammation, oxidative stress, and fibrosis in the renal tissue in mice. Relative mRNA levels of TNF-*α* (a), IL-6 (b), TGF-*β*1 (c), and p47phox (d). GAPDH was used as a housekeeping gene. Data are presented as the mean ± standard deviation, *n* = 4. The same superscript letters indicate no significant difference between groups (*P* > 0.05); significant difference existed between groups that do not have a same superscript letter (*P* < 0.05).

Similar to the changes occurring in SHR, RT-qPCR data (Figure 10) revealed relative TNF-*α*, IL-6, p47phox, and TGF-*β*1 mRNA amounts in the renal tissue in the *Ang II* group were markedly elevated compared with those of the two groups infused saline. The upregulation mediated by Ang II could be inhibited by Rg3 treatment, as the levels of these four mRNAs in the *Ang II* + *Rg3* group were significantly lower than those of the *Ang II* group. These findings indicated Rg3 treatment attenuated inflammation, oxidative stress, and fibrosis caused by Ang II infusion in the renal tissue in mice.

## 4. Discussion

Chronic kidney disease (CKD) represents a growing global health problem [20, 21]. Ang II is a major risk factor for CKD, e.g., hypertensive and diabetic nephropathies. Ang II-associated oxidative stress, inflammation, and fibrosis result in reversible or irreversible pathological changes in the renal tissue, which could progress into end-stage renal disease [21].

To counteract Ang II increase in the renal tissue in early-stage hypertensive or diabetic nephropathy, the expression of ACE2 will be compensatorily upregulated. This self-protective mechanism becomes ineffective as the injury is sustained, and hypertensive or diabetic nephropathy would progress into the decompensation stage [19].

It was reported that human recombinant ACE2 injection could enhance the renal protective effects of ACE2 compensatory upregulation [15, 19]. These protective effects occur even in ACE2-knockout mice [15]. Therefore, it is available to reduce the progression of nephropathy increasing ACE2 levels in the renal tissue.

Rg3 exerts protective effects on various organs, including the heart, kidney and liver [6, 9, 10, 22]. We have demonstrated that the cardioprotective effects of Rg3 are associated with Ang II reduction in the myocardium [10]. Therefore, we aimed to assess whether the protective effects of Rg3 on the kidney are associated with Ang II reduction, and ACE2 levels are raised in the renal tissue.

Rg3, the principal component of Shenyi Capsule, is usually administered orally in clinical, and then it is metabolized by human intestinal bacteria. The metabolic product includes several kinds of ginsenosides [23, 24], such as ginsenoside Rh2 and protopanaxadiol. As Rg3 do not affect the kidneys directly, it is suitable to study the renal protective effects of Rg3 with animal experiments.

We carried out experiments in two models of nephropathy to test the hypothesis mentioned above. The rat model was at the compensation stage, with endogenous Ang II increase in the renal tissue, while the mouse model was at the decompensation stage, with exogenous increase of Ang II. These two experiments were mutually complemental.

The results obtained in both models were consistent. Rg3 reduced Ang II levels in the renal tissue in both models by up-regulating ACE2, and the mRNA levels of TNF-*α*, IL-6, p47phox, and TGF-*β*1 were down-regulated. These situations are similar to the previously reported [15], in which exogenous supplementary ACE2 downregulate Ang II levels.

In that research, upregulating ACE2 attenuated Ang II associated inflammation, oxidative stress and fibrosis in the renal tissue. Inflammatory cytokines such as TNF-*α*, IL-6, IL-1*β*, and CCL-5 are down-regulated in renal tissue, the count of CD3 + cells was cut down. Dihydroethidium fluorescence showed that ACE2 reduced reactive oxygen species (ROS) in renal tissue, which mechanism was reversing NADPH Oxidase Activation. The syntheses of *α*-smooth muscle actin (*α*-SMA) and Collagen I & III were reduced by downregulating TGF-*β*1.

Because Rg3 upregulated ACE2 and downregulated TNF-*α*, IL-6, p47phox, and TGF-*β*1 as well as the research mentioned above, we determine that Rg3 attenuated Ang II associated inflammation, oxidative stress and fibrosis in the renal tissue. Meanwhile, Rg3 was able to reduce the progression of nephropathy in both models.

As there is no relevant report that could be retrieved, it seems that this research is the first to demonstrate that Rg3 upregulates tissue ACE2 levels *in vivo*. However, further investigation is needed to confirm that ACE2 upregulation in the renal tissue is the key mechanism of the renal protective effects of Rg3, and to reveal how Rg3 upregulates them. We might need to use other animals and models, for example, ACE2-knockout mice.

## 5. Conclusion

Overall, the current findings suggested Rg3 attenuates Ang II-associated kidney injury and reduces the progression of nephropathy in animals. The probable mechanism is that Rg3 could reduce Ang II levels in the renal tissue by upregulating ACE2 and attenuate Ang II-induced inflammation, oxidative stress, and fibrosis in the renal tissue.

## Figures and Tables

**Table 1:** Primer sequences of GAPDH, TNF-*α*, IL-6, TGF-*β*1, and p47phox.

Primer names	Sequences
Rats	
GAPDH	Forward: 5′TTGTCAGCAATGCATCCTGC-3′
	Reverse: 5′-CGGCATGTCAGATCCACAAC-3′
TNF-*α*	Forward: 5′-GTCGTAGCAAACCACCAAGC-3′
	Reverse: 5′-TGTGGGTGAGGAGCACGTAG-3′
IL-6	Forward: 5′-TGTATGAACAGCGATGATG-3′
	Reverse: 5′-AGAAGACCAGAGCAGATT-3′
TGF-*β*1	Forward: 5′-ACCTGCAAGACCATCGACATG-3′
	Reverse: 5′-CGAGCCTTAGTTTGGACAGGAT-3′
p47phox	Forward: 5′-GCACTGAAAGGCGGTCCTAT-3′
	Reverse: 5′-TACCCGTGGAGAGAAACCCA-3′

Mice	
GAPDH	Forward: 5′-CCCTTCATTGACCTCAACTACATG-3′
	Reverse: 5′-CTTCTCCATGGTGGTGAAGAC-3′
TNF-*α*	Forward: 5′-GTCGTAGCAAACCACCAAGT-3′
	Reverse: 5′-TGTGGGTGAGGAGCACGTAG-3′
IL-6	Forward: 5′-GTCCTTCAGAGAGATACAGAAACT-3′
	Reverse: 5′-AGCTTATCTGTTAGGAGAGCATTG-3′
TGF-*β*1	Forward: 5′-ACCTGCAAGACCATCGACATG-3′
	Reverse: 5′-CGAGCCTTAGTTTGGACAGGAT-3′
p47phox	Forward: 5′-ACCTGTCGGAGAAGGTGGT-3′
	Reverse: 5′-TAGGTCTGAAGGATGATGGG-3′

## Data Availability

The data used to support the findings of this study are included within the article.

## Supplementary Materials

Supplementary Materials Supplementary Figure 1: the chemical structure of Rg3.
