# Supplementary figures and images for: Ginsenoside Rg3 Attenuates Angiotensin II-Mediated Renal Injury in Rats and Mice by Upregulating Angiotensin-Converting Enzyme 2 in the Renal Tissue
Source: Evid Based Complement Alternat Med. 2019 Nov 29;2019:6741057. doi: 10.1155/2019/6741057 (PMC6915024; doi:10.1155/2019/6741057)

## Supplemental Material

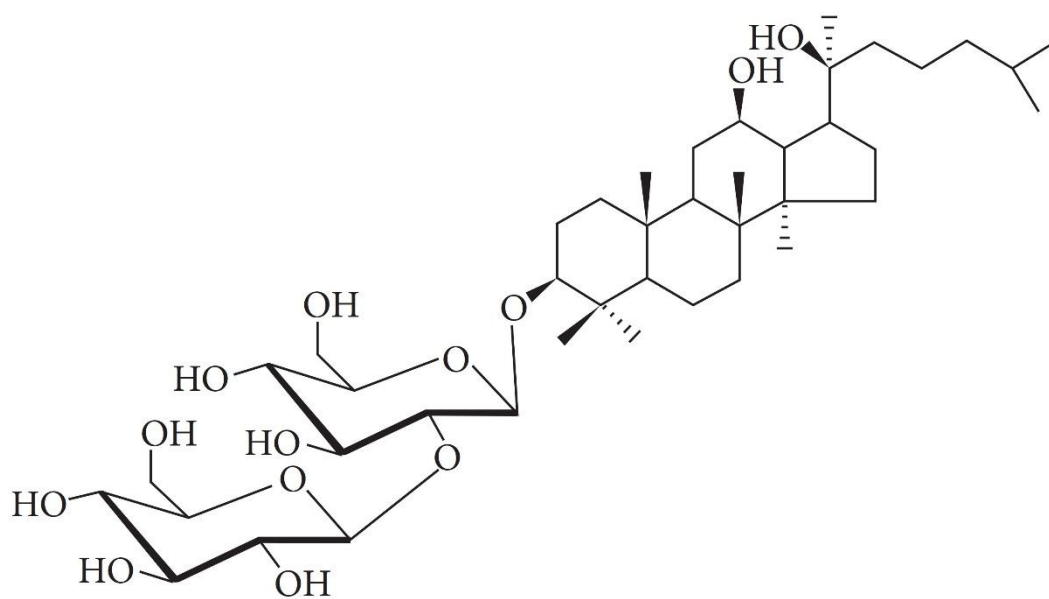

Supplemental Figure 1: The chemical structure of Rg3.

Supplement: Supplementary Materials — Supplementary Figure 1: the chemical structure of Rg3. [file 6741057.f1.pdf]
